# 

**Published:** 1878-08-20

**Authors:** 


### *EDITORIAL AND MISCELLANEOUS*

☞All communications relating to the business of THE RECORD for the years 1877 and 1878, must be addressed DR. R. C. WORD, Managing Editor Southern Medical Record, Atlanta, Ga.

☞Brief and practical communications are solicited on all subjects pertaining to medicine; also reports of cases in practice.

☞Send money by check, postal order or registered letter.

☞Write your name,post-office, county and State plainly.

### NOTICE.

This number of our journal will be sent to many who are not subscribers. We respectfully ask them to try our journal. They will not regret it. The back numbers can be supplied, or may subscribe for six months from the July number.

### TO OUR SUBSCRIBERS.

Please remit your subscriptions for the present year at once. Don't put it off. This is the tightest season of the year in journalism. We greatly need the many small amounts which in the aggregate are very necessary to us. *Please take due notice thereof and govern yourselves accordingly.*

### DERMATOLOGICAL ASSOCIATION.

The American Dermatological Association will be held at Saratoga Springs, New York, on the 27th to the 29th of the present month, August, 1878. Physicians are invited to be present.

Receipted.—Drs. Cheatham & Brother, E A Rowan, R W Rea, W D Hunt, O W Hornsby, J B Rumph, E H Greene, 6 ms.

